# Werner Syndrome-specific induced pluripotent stem cells: recovery of telomere function by reprogramming

**DOI:** 10.3389/fgene.2015.00010

**Published:** 2015-01-29

**Authors:** Akira Shimamoto, Koutaro Yokote, Hidetoshi Tahara

**Affiliations:** ^1^Department of Cellular and Molecular Biology, Graduate School of Biomedical and Health Sciences, Hiroshima UniversityHiroshima, Japan; ^2^Department of Clinical Cell Biology and Medicine, Graduate School of Medicine, Chiba UniversityChiba, Japan

**Keywords:** Werner syndrome (WS), accelerated aging, chromosomal instability, telomere dysfunction, induced pluripotent stem cells (iPSCs), reprogramming, telomerase, premature senescence phenotypes

## Abstract

Werner syndrome (WS) is a rare human autosomal recessive premature aging disorder characterized by early onset of aging-associated diseases, chromosomal instability, and cancer predisposition. The function of the DNA helicase encoded by WRN, the gene responsible for WS, has been studied extensively. WRN helicase is involved in the maintenance of chromosome integrity through DNA replication, repair, and recombination by interacting with a variety of proteins associated with DNA repair and telomere maintenance. The accelerated aging associated with WS is reportedly caused by telomere dysfunction, and the underlying mechanism of the disease is yet to be elucidated. Although it was reported that the life expectancy for patients with WS has improved over the last two decades, definitive therapy for these patients has not seen much development. Severe symptoms of the disease, such as leg ulcers, cause a significant decline in the quality of life in patients with WS. Therefore, the establishment of new therapeutic strategies for the disease is of utmost importance. Induced pluripotent stem cells (iPSCs) can be established by the introduction of several pluripotency genes, including *Oct3/4*, *Sox2*, *Klf4*, and *c-myc* into differentiated cells. iPSCs have the potential to differentiate into a variety of cell types that constitute the human body, and possess infinite proliferative capacity. Recent studies have reported the generation of iPSCs from the cells of patients with WS, and they have concluded that reprogramming represses premature senescence phenotypes in these cells. In this review, we summarize the findings of WS patient-specific iPSCs (WS iPSCs) and focus on the roles of telomere and telomerase in the maintenance of these cells. Finally, we discuss the potential use of WS iPSCs for clinical applications.

## INTRODUCTION

Werner syndrome (WS) is a rare human autosomal recessive disorder characterized by early onset of aging-associated diseases, chromosomal instability, and cancer predisposition (Goto, 1997, 2000). Fibroblasts from patients with WS exhibit premature replicative senescence (Salk et al., 1981b). *WRN*, the gene responsible for the disease, encodes a RecQ-type DNA helicase (Oshima et al., 1996; Yu et al., 1996; Goto et al., 1997; Matsumoto et al., 1997) that is involved in the maintenance of chromosome integrity during DNA replication, repair, and recombination (Shimamoto et al., 2004; Rossi et al., 2010).

WRN is a member of the RecQ helicase gene family, and other members of the family include BLM and RTS/RECQL4, which are mutated in Bloom syndrome (BS) and Rothmund–Thomson syndrome (RTS), respectively (Ellis et al., 1995; Kitao et al., 1999). BS and RTS, along with WS, are characterized by chromosomal instability, due to which RecQ helicases are considered to be the guardian angels of the genome (Shimamoto et al., 2004; Bohr, 2008). There are five members in the RecQ helicase gene family, including RECQL1 (Seki et al., 1994) and RECQL5 (Kitao et al., 1998; Shimamoto et al., 2000), the mutations of which have yet to be identified in human diseases.

Major clinical symptoms of WS include common age-associated diseases, such as insulin-resistant diabetes mellitus, and atherosclerosis. Recent advances in drug therapy for these diseases are available and are known to increase the lifespan of patients with WS. However, there is no effective therapy for intractable features, such as severe skin ulcers leading to a decrease in quality of life (QOL), which is a serious problem in patients with WS. Thus, there is an urgent need to develop a new treatment strategy for this syndrome. Regenerative medicine, such as autologous cell transplantation, could be considered as one of the therapeutic strategies for WS, and a potential choice is the use of patient-specific iPSCs.

Somatic cell reprogramming follows the introduction of several pluripotency genes, including *Oct3/4*, *Sox2*, *Klf4*, *c-myc*, *Nanog*, and *Lin-28*, into differentiated cells such as dermal fibroblasts, blood cells, and others (Takahashi and Yamanaka, 2006; Takahashi et al., 2007; Yu et al., 2007; Aoi et al., 2008; Stadtfeld and Hochedlinger, 2010; Okita and Yamanaka, 2011). During reprogramming, somatic cell-specific genes are suppressed, while embryonic stem cell (ESC)-specific pluripotency genes are induced, leading to the generation of induced pluripotent stem cells (iPSCs) with undifferentiated states and pluripotency (Stadtfeld et al., 2008). Somatic cell reprogramming generates iPSCs characterized by pluripotency and infinite proliferative potential similar to the ESCs, and this technology opens up new possibilities for tailor-made regenerative medicine (Stadtfeld and Hochedlinger, 2010; Okita and Yamanaka, 2011).

Recently, two groups reported the generation of iPSCs from the cells of patients with WS and came to the similar conclusion that reprogramming repressed premature senescence phenotypes in WS cells (Cheung et al., 2014; Shimamoto et al., 2014). They demonstrated the successful reprogramming of cells from patients with WS into iPSCs with restored telomere function and stable karyotypes, suggesting that the induction of the gene encoding human telomerase reverse transcriptase (hTERT) during reprogramming suppresses telomere dysfunction in WS cells lacking WRN. In this review, we summarize the findings of WS patient-specific iPSCs (WS iPSCs) reported in the literature, and focus on the roles of telomere and telomerase in maintenance of these cells. We also review the recent progress in the clinical management of WS and explore stem cell therapy as a new strategy for WS treatment. WS iPSCs will provide opportunities not only for a better understanding of the pathogenic processes and modeling of the complex features of WS, but also for drug screening as well as the discovery and development of a new strategy for its treatment.

## FUNCTION OF WRN HELICASE

**FIGURE 1:**
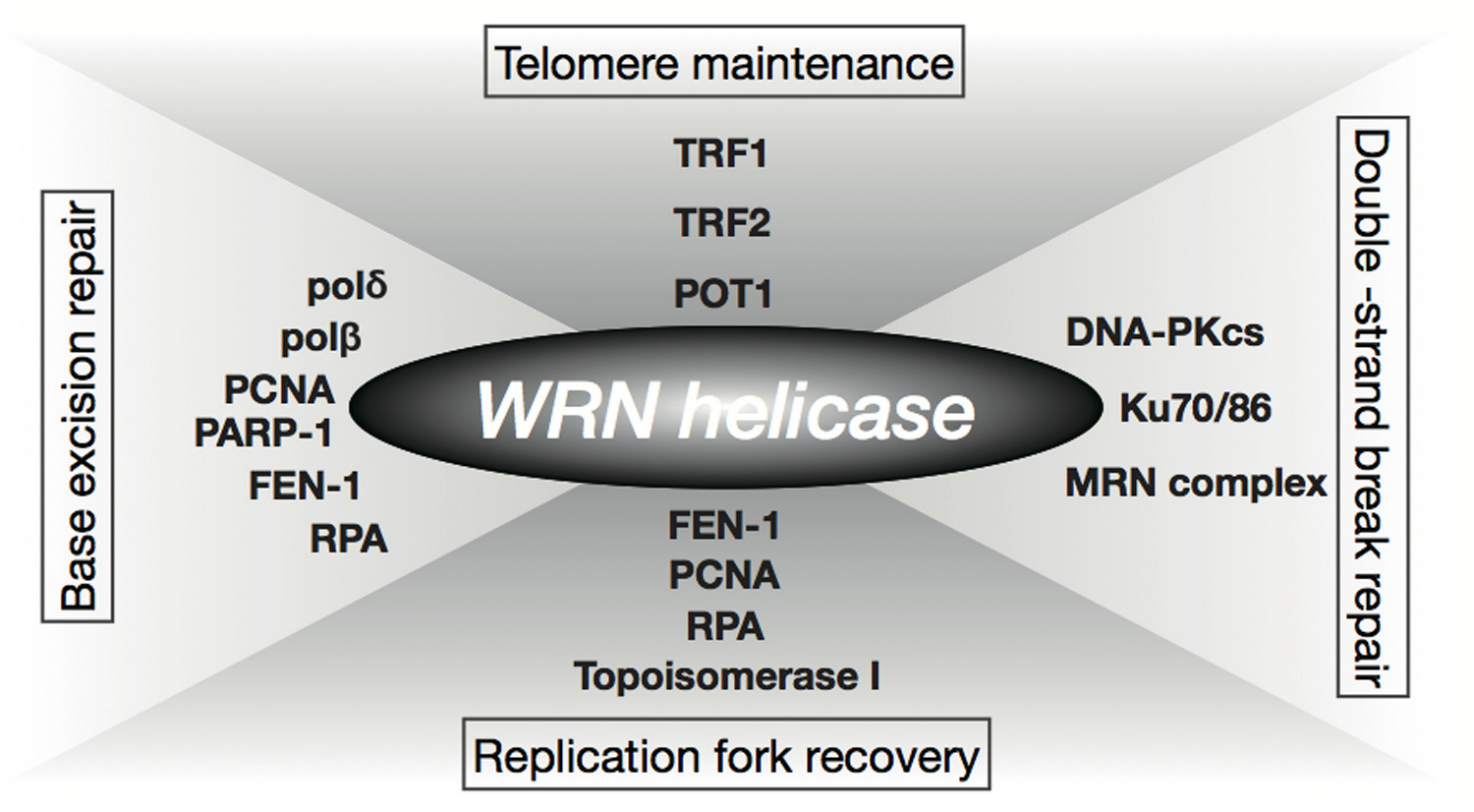
**Function of WRN helicase.** WRN helicase functions through interaction with factors involved in replication (RPA, PCNA, FEN-1, Topoisomerase I), base excision repair (BER; polδ, polβ, PCNA, RPA, FEN-1, PARP-1), double-strand break repair (Ku, DNA-PKcs, Mre11-Rad50-Nbs1 complex), and telomere maintenance, (TRF1, TRF2, POT1).

Prolonged S-phase and reduction in frequency of DNA replication initiation observed in WS cells have implicated the role of WRN helicase in DNA replication (Hanaoka et al., 1983; Poot et al., 1992). The fact that WRN helicase interacts with several factors involved in DNA replication, including RPA, PCNA, FEN-1, and Topoisomerase I, supports this theory (**Figure 1**; Shimamoto et al., 2004; Rossi et al., 2010). WS cells are hypersensitive to a Topoisomerase I inhibitor, camptothecin (Okada et al., 1998; Poot et al., 1999), and WRN nuclear foci induced by the DNA damage caused by camptothecin are co-localized with RPA in the S-phase (Sakamoto et al., 2001). In addition, WRN helicase forms or unwinds the Holliday junction intermediate associated with a regressed replication fork (Sharma et al., 2004; Machwe et al., 2007). These observations suggest that the WRN helicase is involved in the re-initiation of a stalled replication fork. WS cells also show hypersensitivity to 4NQO that induces oxidative damage (Gebhart et al., 1988). Since accumulation of oxidative DNA damage is associated with aging, it is suggested that the WRN helicase is associated with one of the oxidative repair mechanisms, base excision repair (BER), and is known to interact with BER factors, polδ, polβ, PCNA, RPA, FEN-1, and PARP-1 (**Figure 1**; Rossi et al., 2010). Furthermore, the WRN helicase unwinds a BER substrate produced by uracil-DNA glycosylase and AP endonuclease (Ahn et al., 2004). It is also known that the helicase interacts with the double-strand break repair factors Ku, DNA-PKcs, and the Mre11-Rad50-Nbs1 complex, as well as the telomeric DNA protecting proteins, TRF1, TRF2, and POT1 (**Figure 1**; Shimamoto et al., 2004; Rossi et al., 2010). Additionally, Tahara et al. (1997) reported abnormal telomere dynamics in WS lymphoblastoid cell lines (LCLs) with weak or no telomerase activity. These findings suggest that the WRN helicase is involved in telomere metabolism. WRN helicase is shown to resolve Holliday junctions (Sharma et al., 2004), G-quadruplexes formed in telomere G-rich sequences (Mohaghegh et al., 2001), and higher-ordered DNA structures, such as the D-loop (Opresko et al., 2004). These DNA structures formed at telomere ends must be resolved during DNA replication to be accessible to DNA polymerases and telomerase, therefore, WRN helicase might function in the resolution of higher order structures in telomeric DNA.

## ROLES OF TELOMERE IN REPLICATIVE LIFESPAN AND IMMORTALITY

Telomeres, the ends of linear chromosomes in eukaryotes, are ribonucleoprotein-containing specialized structures essential for the protection of chromosomes from a sensing mechanism of double-stranded DNA breaks (Chan and Blackburn, 2004). Mammalian telomeres are composed of TTAGGG repeat sequences, while their specific binding protein complex, shelterin, is composed of the six proteins TRF1, TRF2, RAP1, TIN2, POT1, and TPP1. The chromosome ends are capped by t-loop structures formed by the telomeric DNA and shelterin complex to protect them from DNA damage responses (Palm and de Lange, 2008). In normal human cells, progressive telomere shortening occurs with each successive cell division because of the “end replication problem,” wherein regions of RNA primers involved in lagging strand DNA synthesis at most chromosome ends cannot be replaced with DNA during DNA replication (Harley et al., 1990; Levy et al., 1992). Most of the cells in the human body, such as terminally differentiated cells, have no detectable telomerase activity. Further, tissue stem cells such as hematopoietic stem cells (Vaziri et al., 1994; Allsopp et al., 2001, 2003), epidermal stem cells (Flores et al., 2005), and neural stem cells (Ferron et al., 2004) do not exhibit substantial telomerase activity that can add telomeric repeats sufficient to prevent their chromosomal ends from attrition with successive cell division, which is a major cause of human and other organismal aging (Blasco, 2007). On the other hand, germline stem cells and cancer cells express high levels of telomerase that maintains telomere length sufficient for their immortality (Flores et al., 2006). The human telomerase holoenzyme complex consists of a telomerase reverse transcriptase subunit, hTERT, and a template RNA, TERC, which are the basic components required for catalytic activity. (Egan and Collins, 2012) In addition, it also consists of other accessory proteins, including dyskerin, NHP2, NOP10, and NAF1 required for its assembly and stability (Egan and Collins, 2012). Introduction of hTERT is necessary and sufficient for the activation of telomerase in cells, as other components are already expressed in most normal cells and tissues (Nakayama et al., 1998; Chang et al., 2002). *hTERT* can elongate telomeres, extend the lifespan of normal cells, and immortalize cells such as dermal diploid fibroblasts (Bodnar et al., 1998; Vaziri and Benchimol, 1998; Jiang et al., 1999; Morales et al., 1999). Homologous recombination between telomeres, known as ALT (alternative lengthening of telomeres) is an alternative mechanism for the maintenance of telomere length, and has been observed in subsets of cancer cells, telomerase-deficient ESCs and iPSCs (Dunham et al., 2000; Niida et al., 2000; Wang et al., 2012). These findings indicate that the telomerase-dependent and -independent mechanisms of telomere maintenance are essential for cellular immortality.

## WS FIBROBLASTS EXHIBIT PREMATURE REPLICATIVE SENESCENCE

Intrinsic DNA damage caused by the loss of WRN helicase could activate stress responses leading to cellular senescence. Senescence is defined as a state of permanent cell cycle arrest mediated by the p53-p21^Cip1/Waf1^ and p16^INK4A^-RB pathways. It is one of the tumor suppressor mechanisms exerted in cells that undergo replicative aging with telomere attrition, generation of reactive oxygen species, abnormal proliferation by oncogene activation, and DNA damage activated by DNA damaging agents such as ionizing radiation (Kuilman et al., 2010; Salama et al., 2014). Stress-associated p38 mitogen-activated protein kinase is constitutively activated in WS fibroblasts (Davis et al., 2005). Activation of p38 is known to mediate cellular senescence in the presence of elevated p21 levels (Haq et al., 2002; Iwasa et al., 2003), and p38 inhibitors can suppress premature senescence phenotypes of WS fibroblasts by reducing p21 expression (Davis et al., 2005). These observations indicate that p38 is a major mediator of the reduced replicative lifespan of WS fibroblasts. Meanwhile, activation of p38 also mediates induction of the senescence-associated secretory phenotype (SASP; Freund et al., 2011) that is the hallmark of aging. It is widely accepted that age-associated inflammatory responses contribute to human aging mechanisms (Goto, 2008). WS fibroblasts express inflammatory cytokines (Kumar et al., 1993), and WS is associated with inflammatory conditions responsible for common age-associated diseases, such as atherosclerosis, diabetes, and osteoporosis (Rubin et al., 1992; Murano et al., 1997; Yokote et al., 2004a; Davis and Kipling, 2006). Taken together, these findings suggest that premature replicative senescence with concomitant induction of p21 and SASP, mediated by the activation of p38, could be pathogenic hallmarks of WS.

## TELOMERASE BYPASSES PREMATURE REPLICATIVE SENESCENCE IN WS FIBROBLASTS

**FIGURE 2:**
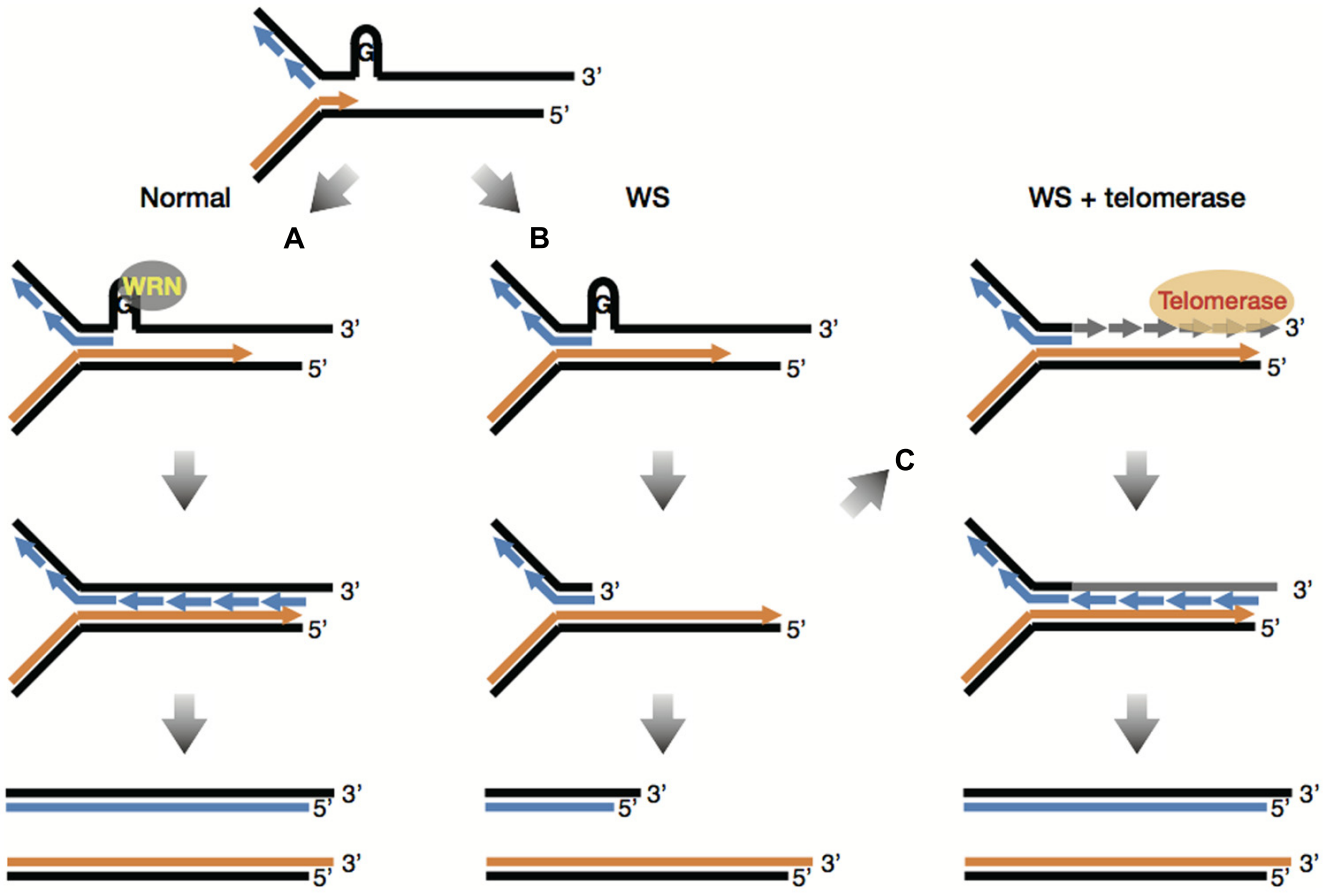
**Telomerase bypasses premature replicative senescence in WS fibroblasts**. **(A)** G-quadruplexes at the lagging telomere are normally unwound by WRN helicase, making it possible to complete replication of lagging strand G-rich telomeres. **(B)** The absence of WRN causes stalled replication forks at the sites of unresolved G-quadruplexes at the lagging telomere, which would produce degradable substrates for factors involved in DNA repair and recombination, leading to accelerated telomere shortening. **(C)** Telomerase can add telomeric DNA “TTAGGG” to lagging telomeres that are lost during replication in WS cells, which overcomes the lack of WRN, enabling complete replication of lagging strand G-rich telomeres. This figure is based on reference (Multani and Chang, 2007).

As mentioned previously, WRN helicase might play an important role in telomere maintenance. This has been verified by Crabbe et al. (2004) wherein, defects in WRN helicase caused impairment of telomeric lagging-strand synthesis and accelerated telomere loss during DNA replication. Moreover, the telomere loss caused by mutation in the WRN gene involved telomere dysfunction such as chromosome end fusions (Crabbe et al., 2007). It is postulated that the absence of WRN causes stalled replication forks at the sites of unresolved G-quadruplexes at the lagging telomere, which would produce degradable substrates for factors involved in DNA repair and recombination, leading to accelerated telomere shortening (**Figures 2A,B**; Multani and Chang, 2007). More importantly, telomerase prevented sister telomere loss (STL) caused by defective telomeric lagging-strand synthesis and suppressed chromosome end fusions in *WRN*-deficient cells (Crabbe et al., 2004, 2007). These results demonstrate that telomerase can provide WS fibroblasts with a complementation effect by adding telomeric DNA “TTAGGG” to lagging telomeres that are lost during replication (**Figure 2C**). Since telomerase is also known to bypass premature replicative senescence in WS fibroblasts (Wyllie et al., 2000), it is suggested that premature senescence in WS cells might be caused by defects in telomeric lagging-strand synthesis followed by telomere loss during DNA replication (Sugimoto, 2014).

## PATHOLOGY IN RECENT WS PATIENTS AND THEIR LIFESPAN

Although WS patients usually grow normally until they reach the late teens, they generally exhibit short stature during adulthood due to impaired maturation. In their 20s and 30s, WS patients start to prematurely develop common age-associated diseases, including cataract, graying of hair and hair loss, atrophic skin, skin ulcers, abdominal fat accumulation, osteoporosis, insulin resistant diabetes mellitus, hypogonadism, atherosclerosis, and cancer (Epstein et al., 1966; Goto, 1997; Mori et al., 2001). Recent increase in life expectancy of patients with WS as well as normal individuals suggest that present-day environment, including diet and medical treatment, might have an effect in delaying and/or improving common age-associated diseases. The clinical review of recent WS case reports was updated in a recent study (Goto et al., 2013). In addition, a nation-wide epidemiological survey was conducted in Japan from 2009 to 2011 to elucidate the current clinical picture of WS, as most patients suffering from this disease are of Japanese origin (Takemoto et al., 2013).

It was found that from 2004 to 2008, patients with WS generally survived until their early 50s; this life expectancy is higher than that in 1966 (below the age of 40), with malignancy and cardiac infarction being the major causes of death (Goto et al., 2013). Several symptoms such as short stature with stocky trunk, bilateral cataracts, graying of hair and hair loss, osteoporosis, and atherosclerosis are still the hallmarks of WS. Skin abnormalities including atrophy, sclerosis, ulcers, pigmentation, and subcutaneous calcification have also been observed recently in most WS patients. Endocrine and metabolic diseases including insulin-resistant diabetes mellitus, hypogonadism, and hyperlipidemia are constantly reported, but not observed in all patients with this disease (Goto et al., 2013).

Our recent epidemiological survey revealed that progeroid changes of the hair, bilateral cataracts, soft-tissue calcifications, and skin abnormalities, including atrophy and intractable ulcers, are the most prominent diagnostic clinical features of WS (Takemoto et al., 2013). Bird-like face and abnormal voice are also the discriminating features of WS. The following features are not observed in all WS patients but are critical symptoms: endocrine and metabolic diseases, such as glucose and/or lipid metabolism abnormalities; bone diseases, such as osteoporosis; atherosclerosis; hypogonadism; short stature; and malignancy (Okabe et al., 2012; Onishi et al., 2012; Takemoto et al., 2013). These endocrine and metabolic symptoms are common age-associated diseases in normal individuals, and recent longevity in the general Japanese population could be attributed to recent advances in medicine. In the same way, the use of current medical care for the treatment of the several critical symptoms of patients with WS might increase their life expectancy (Yokote and Saito, 2008; Goto et al., 2013).

## CURRENT STRATEGIES FOR TREATMENT OF WS

Recent advances in drug therapy for common age-associated diseases are also available for patients with WS. For example, in most of these patients, insulin-resistant diabetes improved by administration of the PPAR-γ agonist pioglitazone that is generally used for the treatment of type 2 diabetes mellitus. In these cases, pioglitazone ameliorated glycemic irregularities and hyperlipidemia as well as impaired insulin sensitivity (Yokote et al., 2004b; Honjo et al., 2008). Insulin-resistant diabetes is also improved by treatment with the dipeptidyl peptidase-4 inhibitor sitagliptin in patients with WS (Kitamoto et al., 2012; Watanabe et al., 2013). Furthermore, hyperlipidemia is one of the predictors of coronary artery disease in WS, and statins have been shown to address this issue in patients with WS (Kobayashi et al., 2000). Since premature senescence in WS cells seem to be caused by accelerated telomere loss during DNA replication (Crabbe et al., 2007), the relationship between telomere and these drugs should be considered in the light of protection against aging. It was reported that a short telomere length is a risk factor for coronary heart disease, which is attenuated when combined with the intake of statins (Brouilette et al., 2007). This may be corroborated by a previous finding which suggests that statins prevent telomere dysfunction caused by the loss of telomere repeat-binding factor, TRF2, in cultured endothelial progenitor cells (Spyridopoulos et al., 2004). A PPAR-γ agonist was reported to increase the expression of TRF2 and prevent apoptosis of endothelial progenitor cells (Gensch et al., 2007). Thus, it is likely that improvement in the condition of patients with WS is associated with the effects of these drugs.

## INCREASED LONGEVITY AND QOL IN WS PATIENTS

As mentioned earlier, recent protocols for drug therapy in patients with WS have led to an improvement in their lifespan. The average life expectancy of patients with WS at the Chiba University hospital has increased by more than 10 years from 1987 to 2007 (Yokote and Saito, 2008), and a most recent record reported that the longest-living patient had survived until the age of 64 (Yokote, Personal communication). This retrospective study revealed that 7 of the 11 living patients with WS after 1997 had a history of taking statins and/or pioglitazone, suggesting that medical procedures, possibly improved by drug development, as well as early detection and early intervention may increase the life expectancy of patients with WS. However, improvement in the QOL of these patients is also imperative, as skin ulcers are known to have a negative effect on it (Goto et al., 2013).

Excruciatingly painful skin ulcers in patients with WS are considered to be caused by multiple factors, including dermal fragility caused by a decrease in connective and fat tissues, a delay in wound healing caused by impaired proliferative ability of dermal cells, and poor circulation associated with diabetes and arteriosclerotic lesions, and are extremely difficult to treat (Yeong and Yang, 2004; Takemoto et al., 2013). Severe ulcers are commonly found in heels, ankles, elbows, and other areas subject to pressure, and can be surgically treated in some cases only (Yeong and Yang, 2004). However, drug therapy including basic fibroblast growth factor spray, hydrocolloid dressing, and PGE1 preparation have little effect on the ulcers in WS, although it is reported that topical platelet-derived growth factor-BB and the endothelin receptor antagonist bosentan has shown some beneficial effects (Wollina et al., 2004; Noda et al., 2011). Most deep and severe leg ulcers with necrosis require amputations (Yeong and Yang, 2004; Goto et al., 2013). In spite of the increase in the average of life expectancy in WS patients due to the recent improvement in drug therapy for common age-associated diseases, the decrease in QOL caused by excruciatingly painful ulcers is still a major problem that needs to be addressed in these patients.

Intractable skin ulcers also include diabetic ulcers, stasis ulcers, arterial ulcers associated with arteriosclerotic obliteration and Buerger’s disease, ulcers associated with connective tissue disease, and radiation-induced ulcers. These ulcers might be treated with debridement ointment under infection control for the enhancement of granulation tissue with vascularization and connective tissue repair (Brem and Lyder, 2004; Sorensen et al., 2004). If the affected area contains necrotic tissue, surgical debridement would be performed followed by skin grafting or flap as required (Sorensen et al., 2004). However, as described above, ulcers in patients with WS heal poorly because of atrophic connective and fat tissues, impaired proliferative ability of dermal fibroblasts, and poor circulation, leading to limited healing of skin grafts and flap as a result of defective granulation tissue formation. At present, there is an urgent need to develop an effective therapeutic strategy for the treatment of severe ulcers in patients with WS.

## TELOMERE REJUVENATION IN iPSCs BY REPROGRAMMING

Induced pluripotent stem cells are similar to ESCs, which are generated from individual somatic cells such as dermal fibroblasts, blood cells, and other cell types by the introduction of several pluripotency genes, including *Oct3/4*, *Sox2*, *Klf4*, *c-myc*, *Nanog*, and *Lin-28* (Takahashi and Yamanaka, 2006; Takahashi et al., 2007; Yu et al., 2007; Aoi et al., 2008; Stadtfeld and Hochedlinger, 2010; Okita and Yamanaka, 2011). Because of their ability to differentiate into various cell types as well as their unlimited proliferative potential, iPSCs, like ESCs, are expected to contribute to regenerative medicine (Takahashi et al., 2007; Stadtfeld and Hochedlinger, 2010). However, unlike ESCs, iPSCs are generated from individual patients, therefore, they can be applied to tailor-made medicine based on syngeneic cell transplantation without allograft rejection (Robinton and Daley, 2012; Lin et al., 2013; Takahashi and Yamanaka, 2013). Moreover, disease-specific iPSCs that can differentiate into multiple cell types can be used to resolve the pathogenic processes of several diseases where cell types available from patients are usually limited to patient-derived lymphocytes and/or fibroblasts.

**FIGURE 3:**
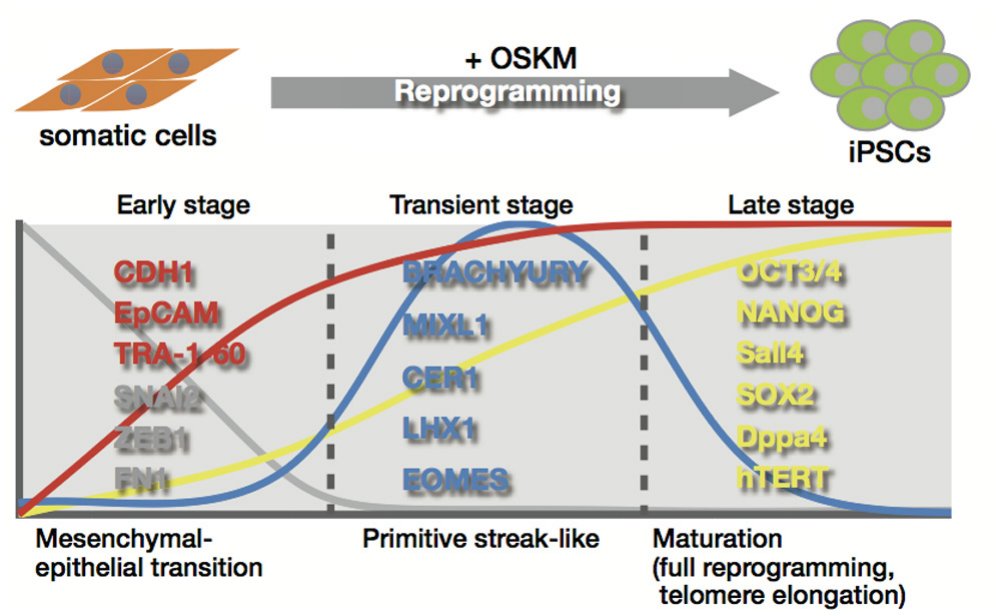
**Telomere rejuvenation by reprogramming.** The reprogramming process is divided into three distinct stages; early stage, transient stage, and late stage. Telomerase activation and telomere elongation occur in the late stage by induction of telomerase reverse transcriptase subunit and the telomerase RNA (TERC) component, which induce telomere rejuvenation similar in length and their epigenetic state to those of ESCs.

The reprogramming process includes several key events that define the mechanism of reprogramming of somatic cells into an ES-like state. One proposed idea separates the process into three distinct phases in human and mouse (**Figure 3**; Samavarchi-Tehrani et al., 2010; Golipour et al., 2012; David and Polo, 2014). In the *Oct3/4*, *Sox2*, *Klf4*, and *c-Myc* (OSKM)-driven reprogramming of mouse embryonic fibroblasts, changes in the expression of genes related to the mesenchymal-to-epithelial transition (MET) are observed in the initiation phase (Mikkelsen et al., 2008; Samavarchi-Tehrani et al., 2010; David and Polo, 2014), along with the loss of mesenchymal cell surface markers, CD44 and Thy1, and a gain of the pluripotency markers, alkaline phosphatase activity, and ESC markers (Stadtfeld et al., 2008; Samavarchi-Tehrani et al., 2010; O’Malley et al., 2013; David and Polo, 2014). MET is also observed in reprogramming of human fibroblasts. Using Tra-1-60 positive intermediate reprogrammed cells, similar events are observed during reprogramming of human fibroblasts (**Figure 3**; Takahashi et al., 2014). MET-associated gene expression change occurs in early phage, where induction of epithelial marker genes such as *CDH1* and *EpCAM*, and suppression of mesenchymal genes including *SNAI2*, *ZEB1,* and *FN1* is observed. In transient stage, intermediate cells transiently express genes related to the primitive streak, including *BRACHYURY*, *MIXL1*, *CER1*, *LHX1,* and *EOMES* (**Figure 3**; Takahashi et al., 2014). These events, along with the later phases, are directly or indirectly regulated though the OSKM transcription network by which chromatin decondensation, loss of suppressive histone modification, DNA demethylation, and gain of active histone modification are directed in the genes to be activated, while a concomitantly opposite regulation is observed in the lineage-specific genes to be inactivated (Apostolou and Hochedlinger, 2013; Buganim et al., 2013; Papp and Plath, 2013). During late stage, activation of the first pluripotency-associated genes, including endogenous *Oct3/4*, *Nanog*, *Sall4*, and *Esrrb*, followed by subsequent activation of *Sox2* and *Dppa4* is essentially required to initiate the transformation into pluripotent cells (**Figure 3**; Stadtfeld et al., 2008; Samavarchi-Tehrani et al., 2010; Buganim et al., 2012; Golipour et al., 2012; David and Polo, 2014; Takahashi et al., 2014). The cells activating the first pluripotency genes successfully shift into late stage from transient stage, leading to the accomplishment of a full reprogramming state that ensures sustained self-renewal ability and differentiation potential. In late stage, successive passages are required to eliminate small differences in gene expression profiles between human iPSCs and hESCs, and to erase epigenetic memory derived from somatic cells used in reprogramming (David and Polo, 2014).

Late stage also involves telomerase activation and telomere elongation that provide somatic cells with infinite proliferative potential (**Figure 3**; Takahashi et al., 2007; Stadtfeld et al., 2008; Marion et al., 2009b; Wang et al., 2012). During the reprogramming process, telomerase is activated by induction of telomerase reverse transcriptase subunit (hTERT in humans) and the telomerase RNA component (TERC; Takahashi et al., 2007; Agarwal et al., 2010; Ji et al., 2013), which are likely to be regulated in stem cells by Wnt/β-catenin signaling with KLF4 and/or TCF4 and OCT3/4 and NANOG, respectively (Agarwal et al., 2010; Wong et al., 2010; Hoffmeyer et al., 2012; Zhang et al., 2012). Although c-Myc is known to induce telomerase activity through direct activation of the hTERT gene (Wang et al., 1998; Wu et al., 1999), it might be less involved in telomerase activation during reprogramming, because OSK of the Yamanaka 4 factors without c-Myc is shown to generate iPSCs with enough telomerase activity (Marion et al., 2009b). hESCs have much longer telomeres with higher expression levels of hTERT and stronger telomerase activity than the differentiated cells (Thomson et al., 1998), and telomerase-dependent telomere maintenance is critical for the growth of mammalian ESCs (Niida et al., 1998). Telomere elongation accompanied by telomerase activation occurs during reprogramming, leading to acquisition of telomeres similar in length to those of ESCs after reprogramming (Marion et al., 2009b). iPSC generation also involves epigenetic alterations to ESC-like states with reduced histone codes associated with heterochromatin, and enhanced transcription at the telomere loci. Elevated frequencies in telomeric sister chromatid exchanges and telomere elongation were observed even when old cells with shortened telomeres were used (Marion et al., 2009b). These observations indicate that telomeres are rejuvenated toward an ESC-like state during reprogramming (**Figure 3**).

However, telomerase-deficient cells with critically shortened telomeres fail to be reprogrammed, suggesting that iPSC generation requires a minimum telomere length to be reprogrammed (Marion et al., 2009b). Critically shortened telomeres resulting from the progression of replicative aging in normal human cells lose the protective function of the chromosomal ends and are recognized as endogenous DNA damage. As a result, dysfunctional telomeres induce DNA damage responses including the activation of ataxia telangiectasia mutated (ATM), ATM- and Rad3-related (ATR), and downstream CHK1 and CHK2 kinases, as well as the phosphorylation of p53, inducing cellular senescence via stimulation of the expression of the cyclin-dependent kinase inhibitor (CDKI) p21 (d’Adda di Fagagna et al., 2003; Herbig et al., 2004; Deng et al., 2008). It has been shown that the activation of p53 significantly suppresses reprogramming efficiency, known as the reprogramming barrier (Hong et al., 2009; Kawamura et al., 2009; Li et al., 2009; Utikal et al., 2009), while suppression of p53 improves the reprogramming efficiency in cells with critically shortened telomeres (Marion et al., 2009a). These findings demonstrate that activation of telomerase during reprogramming plays a pivotal role not only in telomere elongation with chromatin state characteristic of ESCs, but also in the restoration and maintenance of the protective functions of the telomere at the chromosomal ends, in order to suppress DNA damage responses.

## iPSCs AS A POTENTIAL STRATEGY FOR WS TREATMENT

As described above, skin ulcers in patients with WS heal poorly, and so far no effective therapy has been developed to treat them or the other symptoms associated with WS. Thus, there is an urgent need to develop a new treatment strategy in order to improve the health and QOL of patients with WS. Understanding the molecular basis and development of therapeutics requires an appropriate disease modeling system. Primary cells from affected tissues of these patients are required for better understanding of the pathogenic processes and complex features involved with this disease. However, their use is usually limited to patient-derived lymphocytes and/or fibroblasts, which are difficult to propagate in culture for extended periods of time. Thus, regenerative medicine such as autologous cell transplantation could be used as a therapeutic strategy for WS, which provides cells with high proliferative ability and differentiation potential in large quantities over a long period.

The skin, composed of epidermis and dermis, is one of the main affected tissues in WS. A recent study demonstrated that hESCs can generate a homogeneous population of epithelial cells expressing postnatal keratinocyte markers in squamous epithelia, and these hESC-derived keratinocytes could reconstitute a functional pluri-stratified epithelium (Guenou et al., 2009). On the other hand, human epidermal keratinocytes can reconstitute stratified epithelium in culture, and it is known that expanded culture of epidermal stem cells from a tiny skin biopsy can cover the whole body surface of an individual, because of the high proliferative potential of these cells (De Luca et al., 2006), thus raising the argument as to whether pluripotent cell-derived epithelium can be used for clinical purposes (Pellegrini and Luca, 2009). In the case of WS, as the regenerative potential of adult skin cells is expected to be hampered due to their impaired proliferative ability, the concerns over premature senescence phenotype in cells from these patients might be eliminated by the development of rejuvenated resources. Patient-specific iPSCs might be a potential candidate that can meet these requirements. The epoch-making invention of iPSCs has the potential to bring innovation to regenerative medicine as well as drug discovery, as these cells are known to possess the ability to differentiate into all cell types, including those belonging to the skin, hair root, blood vessel, bone, and pancreatic islets.

## GENERATION OF iPSCs FROM WS PATIENT CELLS

During the reprogramming process, both telomere and telomerase play protective roles at chromosomal ends against DNA damage responses, causing a reprogramming barrier (Marion et al., 2009a,b). Fibroblasts from patients with WS exhibit premature senescence caused by accelerated telomere loss during DNA replication (Salk et al., 1981b; Crabbe et al., 2007). Thus, it is interesting to note that forced expression of the telomerase catalytic gene hTERT in WS fibroblasts bypassed the phenotype, raising the question as to whether WS fibroblasts can be reprogrammed into iPSCs. In addition, there are doubts as to whether WS iPSCs, if successfully generated, can maintain hESC-like characteristics during long-term culture. Inconsistent consequences of the generation of patient-specific iPSCs from dyskeratosis congenita (DKC), another disease involving telomere abnormalities, have been reported (Agarwal et al., 2010; Batista et al., 2011). Batista et al. demonstrated that DKC iPSCs presented with progressive telomere shortening and loss of self-renewal ability in long-term culture (Batista et al., 2011). Therefore, it is important to evaluate the properties of iPSCs derived from the cells of the patient with telomere dysfunction over the long term. The findings from a recent study by Cheung et al. demonstrating the successful generation of disease-specific iPSCs from cells of patients with WS were in accordance with one of our current works, leading us to believe that reprogramming repressed premature senescence phenotypes in WS cells (Cheung et al., 2014; Shimamoto et al., 2014).

**FIGURE 4:**
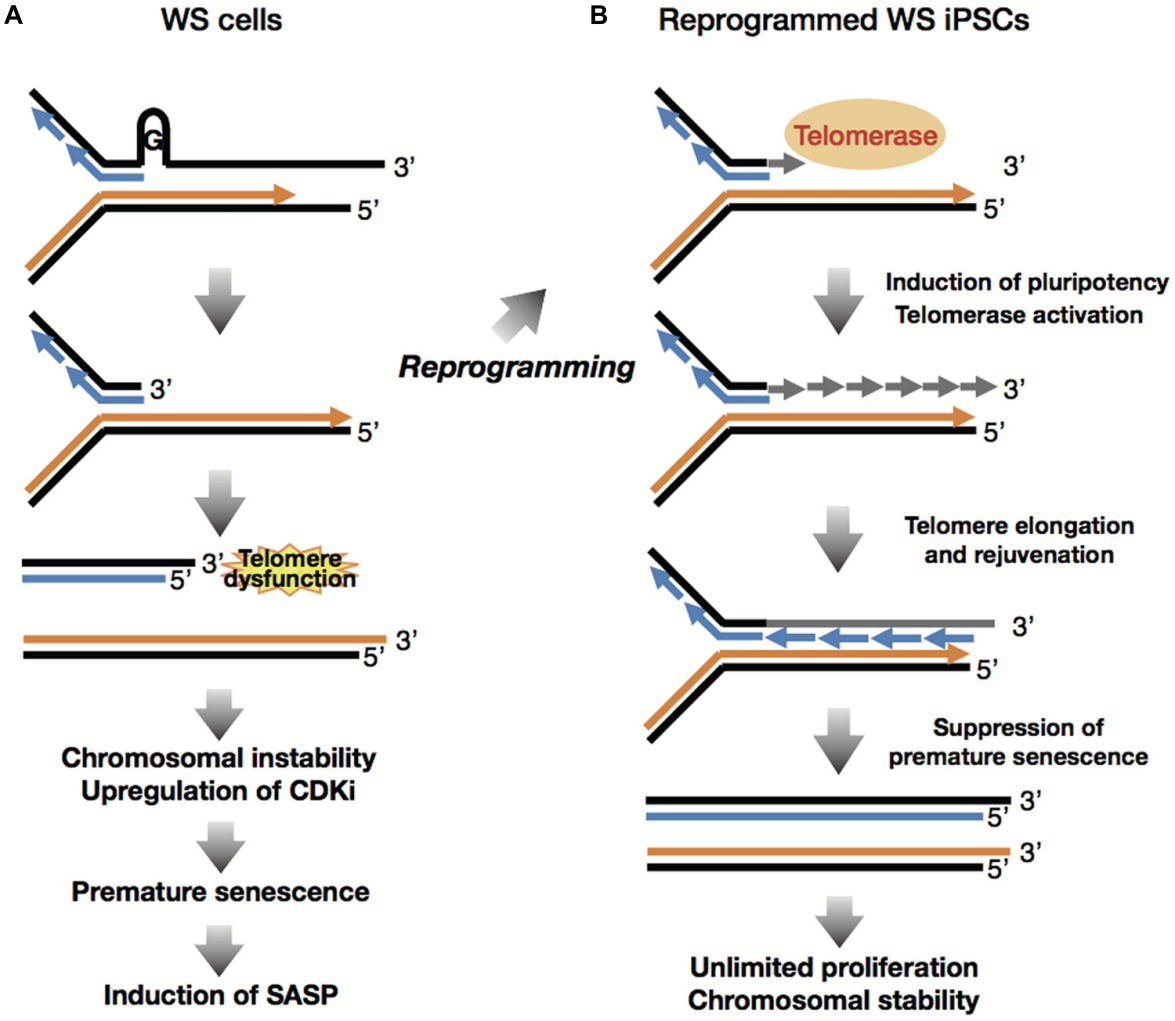
**Reprogramming suppresses premature senescence phenotypes. (style="color:gray" A)** Accelerated telomere shortening during replication induces telomere dysfunction and DNA damage response at the lagging telomere, leading to induction of premature senescence and SASP following chromosomal instability and upregulation of CDK inhibitors. **(B)** Reprogramming involves induction of pluripotency and global epigenetic alteration toward ESC-like state, which suppress senescence phenotypes including CDKi and SASP. Reprogramming also overcomes the lack of WRN through activation of telomerase, consequently inducing telomere elongation and cellular immortality.

WS iPSCs were generated from the patient’s fibroblasts and were quite similar to normal iPSCs in their characteristics as pluripotent stem cells, including their hESC-like morphology, expression of pluripotency genes, and hESC-specific surface markers, global gene expression profiles, embryoid body (EB) formation and subsequent differentiation into three embryonic germ layers, and teratoma formation. The WS iPSCs maintained their telomeres with reactivation of endogenous telomerase by induction of hTERT as well as other components of telomerase, such as TERC and DKC1, and were sustained in culture for more than 35 (Cheung et al., 2014) and 150 passages (Shimamoto et al., 2014) without morphological changes and loss of growth capacity. These observations indicate that induction levels of telomerase activity during reprogramming are sufficient for generation and subsequent cloning and maintenance of iPSCs from WS fibroblasts (**Figure 4**).

## REPROGRAMMING SUPPRESSES PREMATURE SENESCENCE PHENOTYPES AND GENOMIC INSTABILITY OF WS FIBROBLASTS

Expression levels of senescence-associated genes including the CDKIs as well as the SASP factors, were compared between WS fibroblasts and WS iPSCs, because it is widely accepted that age-associated inflammatory responses, including SASP, contribute to human aging mechanisms (Goto, 2008). The results demonstrated that in addition to the CDKI genes *p21^Cip1/Waf1^* and *p16^INK4A^*, the SASP genes such as *IL-6*, *gp130*, *IGFBP5*, *IGFBP7*, *ANGPTL2*, and *TIMP1* were highly expressed in cells of patients with WS as compared with PDL-matched normal fibroblasts. However, the expression levels of the same genes were suppressed in their iPSC derivatives to the level generally seen in normal iPSCs. These observations revealed that reprogramming suppresses and rejuvenates the premature aging phenotypes of WS fibroblasts (**Figure 4**; Shimamoto et al., 2014).

WS is characterized by genomic instability and chromosomal aberrations, including translocations, inversions, and deletions that have been observed during culture of patient-derived cells (Salk et al., 1981a). As the generation and subsequent maintenance of iPSCs involve extensive cell division, WS iPSCs may acquire additional chromosomal abnormalities during the process. In one of our recent works, we performed a chromosomal profiling analysis which indicated karyotype stability in WS iPSCs in long-term culture (Shimamoto et al., 2014). We performed G-banding stain and multicolor fluorescence *in situ* hybridization, and showed that 3 of 6 WS iPSC clones had the same karyotypes as their parental cells after approximately 100 passages, suggesting that karyotypes of WS cells are stabilized following reprogramming (**Figure 4**; Shimamoto et al., 2014). Normal human iPSCs are known to acquire genomic instability with high incidence of additions, deletions and translocations (Martins-Taylor et al., 2011; Taapken et al., 2011). Thus, given the genomic instability of WS cells, these data reveal the unexpected maintenance of chromosomal profiles in WS iPSC clones during long-term culture, indicating the possibility of its application clinically, although general risk factors, including genetic and epigenetic abnormalities (Gore et al., 2011; Hussein et al., 2011; Lister et al., 2011; Pera, 2011) and the potential for tumorigenicity (Kiuru et al., 2009; Ben-David and Benvenisty, 2011) and immunogenicity (Zhao et al., 2011; Araki et al., 2013) must be taken into consideration.

## RECAPITULATION OF PREMATURE SENESCENCE PHENOTYPES IN DIFFERENTIATED CELLS

**FIGURE 5:**
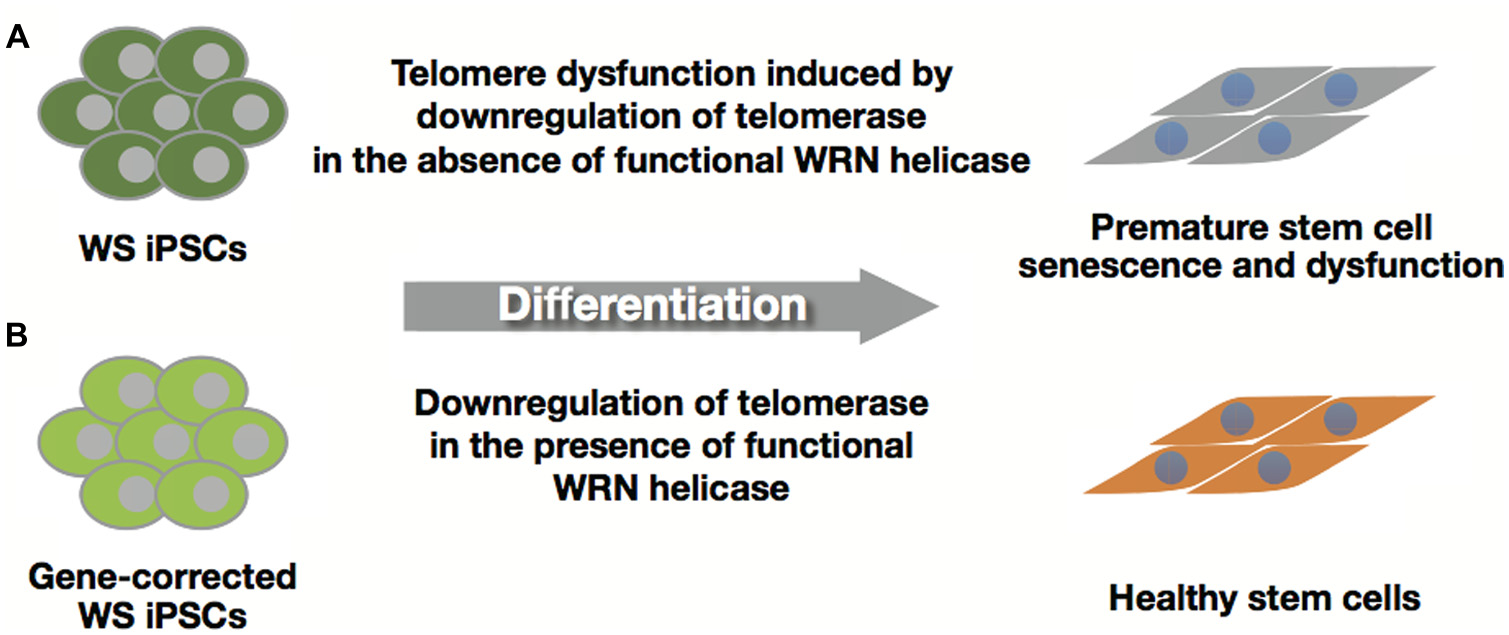
**Healthy stem cells from gene-corrected WS iPSCs. (A)** WS iPSC-derived stem cells with insufficient levels of telomerase activity induce premature senescence and stem cell dysfunction. **(B)** Gene-corrected WS iPSCs can produce healthy stem cells by suppressing premature senescence due to functional WRN helicase.

Differentiated cells including mesenchymal stem cells (MSCs) and other cell types derived from WS iPSCs were examined to determine their roles as models of WS, because the WS iPSCs do not exhibit any of the characteristic features of the syndrome. Cheung et al. demonstrated premature senescence of WS MSCs with elevated expression levels of p53, p21, and p16; accelerated telomere shortening; and impaired telomeric lagging-strand synthesis that causes telomere loss and dysfunction (Cheung et al., 2014). They also showed that WS iPSC-derived neural progenitor cells (NPCs) expressing telomerase activity maintained telomere and proliferative capacity with NPC phenotypes, and treatment with telomerase inhibitor was seen to decrease growth and increase the incidence of γH2AX in WS NPCs. These results, together with the fact that hTERT rescues premature senescence and telomere dysfunction, suggest that premature senescence in WS MSCs is due to insufficient levels of telomerase activity downregulated during differentiation (**Figure 5A**). Thus, adequate telomerase activity could maintain tissue stem cell function in WS (Cheung et al., 2014).

Differentiated cells derived from WS iPSC EBs were also examined for their growth defects. We found that the cells underwent premature senescence with a higher rate of SA-β-gal positive cells, upregulation of p21 concomitantly with downregulation of hTERT and induction of SASP genes (**Figure 5A**; Shimamoto et al., 2014). Since EB-derived differentiated cells include a variety of cell types originating from the three germ layers, these results suggest that EB-mediated iPSC differentiation could provide a simple and rapid method for the identification of cell lineages other than the MSCs in WS.

*ATM*, a causative gene for premature aging syndrome ataxia telangiectasia (AT) is required for the maintenance of stem cells including hematopoietic and spermatogonial stem cells (Ito et al., 2004; Takubo et al., 2008). Furthermore, the pathophysiology of AT might be associated with dysregulation of the reservoir of adult stem cell populations (Wong et al., 2003). Recent findings have shown dysfunction of vascular smooth muscle cells (VSMCs) and their progenitor SMCs in Hutchinson–Gilford Progeria syndrome (HGPS; Liu et al., 2011; Zhang et al., 2011). Taken together, these findings suggest that premature aging syndromes including WS, AT, and HGPS are stem cell dysfunction-associated diseases.

## GENE-CORRECTED WS iPSCs AND ITS CLINICAL APPLICATION

Autologous cell transplantation can be selected as one of the therapeutic strategies for treating the intractable symptoms of WS, including skin ulcers. Clinical application of iPSCs requires unlimited proliferative ability and differentiation potential into various cell types with healthy conditions that could replace the affected area. Although WS iPSCs are almost indistinguishable from normal iPSCs in many aspects, differentiated cells from WS iPSCs manifest premature aging phenotypes (**Figure 5A**; Cheung et al., 2014; Shimamoto et al., 2014). Thus, gene-corrected WS iPSCs could offer a unique treatment strategy for patients with WS. Recent progress in gene therapy and genome engineering technology provides powerful tools for genome editing, including zinc-finger nucleases (ZFNs), transcription activator-like effector nucleases (TALENs), and RNA-guided engineered nucleases derived from the bacterial clustered regularly interspaced short palindromic repeat (CRISPR)-Cas system (Kim and Kim, 2014; Li et al., 2014). This technology could be used for correction of the disease-specific mutations in iPSCs by gene targeting (Yusa et al., 2011; Suzuki et al., 2014) and establishment of disease-specific iPSCs from wild-type iPSC lines (Soldner et al., 2011). Because WS is inherited in an autosomal recessive manner, a single gene-targeting event of specific mutations in WRN loci might be sufficient for recovery from WS, which could be confirmed by examining differentiated cells such as MSCs for the restoration of telomere dysfunction and premature growth defects (**Figure 5B**). In addition to the safety of iPSCs (Okano et al., 2013), evaluation of whole-genome sequencing and epigenomic analysis will be needed before their clinical application because WS patient cells are reported to have chromosomal aberrations including translocations, inversions, and deletions (Salk et al., 1981a). Furthermore, the differentiation potential and corrected phenotypes in gene-corrected WS iPSCs must be warranted for their clinical use. For example, reconstituted epithelium might be clinically applicable for skin ulcers after finding evidence that gene-corrected WS iPSC-derived keratinocytes could form functional pluri-stratified epithelium with fibroblast-containing fibrin dermal matrix *in vivo* (Guenou et al., 2009).

## CONCLUSION

Recent findings including ours, demonstrate that reprogramming bypasses premature senescence and suppresses genomic instability in WS cells, leading to sustained undifferentiated states with the ability to differentiate into three embryonic germ layers over the long term. It is noteworthy that WS iPSCs exhibited stable chromosomal profiles, and this unexpected property might be achieved by the expression of the endogenous telomerase gene induced during reprogramming. As normal iPSCs exhibited higher expression levels of WRN protein than normal fibroblasts, WRN helicase might have a role in chromosomal stability as well as telomere maintenance in iPSCs. Thus, thorough safety tests, especially concerning genetic and epigenetic abnormalities and the potential for tumorigenicity, will be necessary before the clinical application of these cells. The use of WS iPSCs will enhance our understanding of the pathogenic processes and modeling of complex features associated with WS. In addition, it can provide opportunities for drug screening and the discovery and development of new strategies for the treatment this disease. Finally, although challenges and concerns remain regarding the general safety and risk of iPSCs as well as the WS-specific defects, a recent clinical trial using patient-specific iPSCs at the RIKEN Center for Developmental Biology (CDB) will encourage and promote stem cell research toward clinical application (Reardon and Cyranoski, 2014), at the same time, we need to consider the results of the RIKEN CDB clinical trial in a calm manner.

## Conflict of Interest Statement

The authors declare that the research was conducted in the absence of any commercial or financial relationships that could be construed as a potential conflict of interest.
